# News and Events of the Week

**Published:** 1911-02-04

**Authors:** 


### News and Events of the Week.

Lord Ilkeston, M.D., Sir H. Bryan Donkin, M.D., Dr. R. Caton, of Liverpool, and Dr. J. Phillips, all Fellows of the Royal College of Physicians, have been elected Members of Council.

Mr. Johnson, M.R.C.S., a medical practitioner of Cambridge, was cut badly about the face last Sunday in a motor-car accident, in which three cars collided on the Newmarket Road during a fog. Mr. Johnson's car was wrecked.

The Lettsomian Lectures of the Medical Society of London will be delivered by Mr. Haslam, F.R.C.S., on February 6 and 20 and March 6, at 11 Chandos Street, Cavendish Square, W. The subject will be "A Review of the Operations for Stone in the Male Bladder."

A telegram from Kharbin, published in a recent issue of the *Times*, stated that Mme. Lebedeff, a lady doctor, has died of the plague, as well as a medical student and a hospital assistant. Six doctors and fifty hospital assistants have been sent from Siberia to reinforce the medical staff in the infected districts.

The balance-sheet of the London County and Westminster Bank again shows the steady prosperity of this respected house, the gross profits for the year, after bad debts and contingencies had been provided for, amounting to £2,820,776. The balance carried forward has now risen to £156,229. The balance-sheet can be studied in detail in our advertisement columns, where it is published in full.

Four lectures on "Plague" will be delivered on Tuesday, February 14; Wednesday, February 15; Thursday, February 16; and Friday, February 17, 1911, by F. M. Sandwith, M.D., Gresham Professor of Physic. The lectures will be delivered at the City of London School, Victoria Embankment, E.C. (three minutes' walk from Blackfriars Bridge), and are free to the public, and will begin each evening at six o'clock.

The following is the course of lectures and demonstrations at the Hospital for Sick Children, Great Ormond Street, W.C., for the session January to March, 1911, free to medical practitioners, on Thursdays, at 4 p.m. : February 9, Demonstration of Surgical Cases, Mr. Kellock; February 16, Some further Complications of Rheumatism, Dr. Poynton; February 23, Pathological Demonstration, Dr. Frew; March 2, Demonstration of Medical Cases, Dr. Batten; March 9, Conjunctivitis in Children, Mr. Coats; March 15, Coxavara, Mr. Addison; March 23, Some Affections of the Newborn, Dr. Still; March 30, Appendicitis in Children, Mr. Fairbank.

Our excellent contemporary, the *Medical Officer*, has introduced a new feature in the form of a supplement containing concise information as to recent work in bacteriology and general parasitology and allied subjects in their relationship to pathology and hygiene. It is intended in addition to publish from time to time articles dealing with special subjects of current interest, or in which recent advances in knowledge have been made. The new feature will prove of interest to the general practitioner as well as to that branch of the body medical for which the journal is primarily intended. The new supplement will be under the capable supervision of Mr. A. G. R. Foulerton, F.R.C.S., and will appear at least once in each month.

Sir Francis Champneys has been appointed to represent the Royal College of Physicians on the Central Midwives Board.

The anniversary dinner of the Medical Society of London will take place at the Whitehall Rooms, Hotel Metropole, on Wednesday, March 8 next, at 7.30 p.m.

At a Hertfordshire county meeting, Lord Clarendon, Lord-Lieutenant, in the chair, it was unanimously resolved, on the motion of Sir Alfred Reynolds, High Sheriff: "That the memorial to his late Majesty King Edward VII. shall take the form of an endowment proportionately of each hospital in the county, including the Hertfordshire Convalescent Homes, and that a tablet shall be fixed in each hospital stating the form the memorial has taken, and that a portrait of his late Majesty be fixed over the same."

Details of the census of the French population for 1906 are just to hand, from which it is shown that 20,720,879 persons exercise a trade or profession and form the *active population*. Of this number 13,027,467 are males and 7,693,412 females, so that women form more than a third of the active population. With the exception of domestic service the men, however, are more numerous in all the chief professions. In 1866 the active population was 40 per cent. of the whole, in 1896, 49 per cent., and in 1906, 53 per cent. Thus the proportion of workers in the population has sensibly increased of late, and within forty years male workers have increased by two and a-half and female workers by over three millions. There has, however, been a drop since 1866 of 274,441 in the number of people engaged in domestic service.

If official statistics are to be credited, the consumption of beer is rapidly decreasing in Germany. During 1909 there was a falling-off in the amount consumed of no less than four and a-half litres per head of population. In 1908 the decrease was even greater. There is also a gradual diminution in the number of breweries in the country. In 1906 these numbered 6,035, in 1907 there were 5,792, in 1908 only 5,590 were left, and in 1909 there was a still greater decrease to 4,768. An extraordinary fact, however, is that the quantity of malt used in the manufacture of beer has decreased out of all proportion to the drop in consumption of the liquid itself. It would seem, therefore, that beer of a lighter quality is being brewed. What with the French ceasing to drink wine, the Germans drinking less beer, and the Chinese becoming tired of tea, it looks as though the day of the mineral-water manufacturer had come to stay!

The following are the arrangements for next week at at West London Postgraduate College: At 10 a.m., February 6 and 9, Demonstration by Surgical Registrar; at 10 a.m., February 8, Gynæcological Demonstration; at 11.30 a.m., February 7, Demonstration of Minor Operations; at 12 noon, February 6, Pathological Demonstration; at 12 noon, February 9, Lecture, Dr. Grainger Stewart, Practical Medicine. Lectures at 5 p.m.: February 6, Mr. Dunn, The Tragedies of Ocular Disease; February 7, Dr. Seymour Taylor, Rheumatism in Childhood; February 8, Dr. Grainger Stewart, Disseminated Sclerosis with Cases; February 9, Dr. Grainger Stewart, Early Cases of General Paralysis; February 10, Dr. Pritchard, Practical Medicine.

